# Relationship between serum calcium or phosphate levels and mortality stratified by parathyroid hormone level: an analysis from the MBD-5D study

**DOI:** 10.1007/s10157-020-01879-8

**Published:** 2020-03-31

**Authors:** Shinji Asada, Keitaro Yokoyama, Chisato Miyakoshi, Shingo Fukuma, Yuichi Endo, Michihito Wada, Takanobu Nomura, Yoshihiro Onishi, Masafumi Fukagawa, Shunichi Fukuhara, Tadao Akizawa

**Affiliations:** 1 Medical Affairs Department, Kyowa Kirin Co., Ltd, Otemachi Financial City Grand Cube, 1-9-2 Otemachi, Chiyoda-ku, Tokyo, 100-0004 Japan; 2 Division of Nephrology and Hypertension, Department of Internal Medicine, Jikei University School of Medicine, Tokyo, Japan; 3 Jikei University Harumi Triton Clinic, Jikei University School of Medicine, Tokyo, Japan; 4 Department of Healthcare Epidemiology, School of Public Health, Faculty of Medicine, Kyoto University, Kyoto, Japan; 5 Department of Pediatrics, Kobe City Medical Center General Hospital, Kobe, Japan; 6 Institute for Health Outcomes and Process Evaluation Research (iHope International), Kyoto, Japan; 7 Human Health Sciences, Kyoto University Graduate School of Medicine, Kyoto, Japan; 8 The Keihanshin Consortium for Fostering the Next Generation of Global Leaders in Research (K-CONNEX), Kyoto, Japan; 9 Division of Nephrology, Endocrinology and Metabolism, Tokai University School of Medicine, Isehara, Japan; 10 Division of Nephrology, Department of Medicine, Showa University School of Medicine, Tokyo, Japan

**Keywords:** Calcium, Chronic kidney disease–mineral and bone disorder, Hemodialysis, Parathyroid hormone, Phosphate, Mortality

## Abstract

**Introduction:**

There is limited evidence about the association between calcium and phosphate levels and mortality stratified by intact parathyroid hormone (iPTH) level.

**Methods:**

We investigated whether differences in iPTH level affect the relationship between calcium and phosphate levels and all-cause mortality in hemodialysis patients with secondary hyperparathyroidism (SHPT). Calcium and phosphate levels were categorized as low (< 8.5 mg/dL, < 4.0 mg/dL), medium (≥ 8.5–< 9.5 mg/dL, ≥ 4.0–< 7.0 mg/dL), and high (≥ 9.5 mg/dL, ≥ 7.0 mg/dL), respectively. iPTH levels were grouped into < 300 or ≥ 300 pg/mL. Adjusted incidence rate ratios (aIRRs) were analyzed by weighted Poisson regression.

**Results:**

For calcium, patients with higher iPTH (≥ 300 pg/mL) had significantly higher all-cause mortality rates in the high than in the medium category (aIRR 1.99, 95% confidence interval [CI] 1.16–3.42), and tended to have a higher mortality rate in the low category (aIRR 2.04, 95% CI 0.94–4.42). Patients with lower iPTH (< 300 pg/mL) had higher mortality rates in the high than in the medium category (aIRR 1.65, 95% CI 1.39–1.96). For phosphate, the mortality rate was significantly higher in the high than in the medium category in patients with higher and lower iPTH (aIRR 3.23, 95% CI 1.63–6.39 for iPTH ≥ 300 pg/mL; aIRR 1.58, 95% CI 1.06–2.36 for iPTH < 300 pg/mL).

**Conclusion:**

High calcium and phosphate levels were associated with increased risk of mortality irrespective of iPTH level.

**Electronic supplementary material:**

The online version of this article (10.1007/s10157-020-01879-8) contains supplementary material, which is available to authorized users.

## Introduction

Chronic kidney disease (CKD) may involve secondary hyperparathyroidism (SHPT) characterized mainly by abnormal metabolism of calcium and phosphate, and increased production and secretion of parathyroid hormone (PTH). The resulting osteoporosis and blood vessel calcification due to the abnormal mineral metabolism influences the onset of cardiovascular events that worsen the patient’s prognosis [1]. In the management of abnormal mineral metabolism, patients are treated according to target control values of phosphate, calcium, and PTH levels [2, 3].

Associations between elevated phosphate and calcium levels and increased mortality rates have been shown in the Worldwide Dialysis Outcomes and Practice Pattern Study (DOPPS) in hemodialysis patients [4] and in a study by Block et al. that reported real-world findings in the United States [5]. Similar findings were reported from Japanese studies in dialysis patients with SHPT (the Mineral and Bone Disorder Outcomes Study for Japanese CKD Stage 5D Patients [MBD-5D]) [6] and an exhaustive survey in maintenance hemodialysis patients [7]. In addition, Tentori et al. evaluated the relationships between calcium and phosphate levels and mortality rates in a population with an intact PTH (iPTH) level of ≥ 300 pg/mL and < 300 pg/mL, reporting that in the subpopulation with iPTH ≥ 300 pg/mL, the mortality rate was higher in patients with high calcium levels, suggesting a U-shaped relationship between phosphate level and mortality rate, and that in the subpopulation with iPTH < 300 pg/mL, the mortality rate tended to be higher in patients with low calcium levels [4]. However, the relationships between phosphate and calcium levels and mortality rates depending on SHPT severity have not been thoroughly assessed in Japan.

We therefore aimed to explore any associations between SHPT severity and calcium and phosphate levels and mortality rates with iPTH as an index using a marginal structural model in the MBD-5D study, a prospective observational study in SHPT patients on maintenance hemodialysis.

## Methods

### MBD-5D study

The MBD-5D study was a multicenter, prospective, case-cohort study following maintenance hemodialysis patients with SHPT [8]. A total of 8229 patients were registered from 86 facilities in Japan, and followed for 3 years (from January 2008 to December 2010). Data for prescribed drugs and mineral and bone disorder (MBD)-related serum markers (serum calcium, phosphate, and iPTH) were recorded every 3 months and data for other time-dependent variables were recorded every 6 months.

Of the total number of patients in the cohort, 40% were randomly selected into the subcohort (*n* = 3276), for which data were collected prospectively. Data from patients who died and had not been included in the subcohort were collected retrospectively. The study protocol was approved by the central ethics committee at Kobe University’s School of Medicine (No. 754). Informed consent was not mandatory according to the ethical guidelines for epidemiological research in Japan. The study is registered at ClinicalTrials.gov (NCT00995163).

### Participants

The target population of the MBD-5D study was patients with SHPT who had received maintenance hemodialysis. Patients with SHPT were defined as those who had iPTH ≥ 180 pg/mL (according to the Japanese guidelines at that time [3], such patients needed treatment to lower iPTH levels), or those who were treated with intravenous calcitriol or maxacalcitol and/or an oral vitamin D receptor activator (VDRA; falecalcitriol). All the eligible patients receiving maintenance hemodialysis at one of the participating facilities as of January 2008 were enrolled. Patients who had been receiving hemodialysis for less than 3 months were excluded. Although the study protocol did not specify the management of patients, we assumed that these patients had been managed according to Japanese guidelines.

### Exposures, outcomes, and covariates

The primary outcome was all-cause mortality. The secondary outcome was cardiovascular mortality, which was defined as death due to cerebrovascular disease, heart failure, myocardial infarction, sudden death, arrhythmia, aortic disease, or other cardiovascular disease. The MBD-related serum markers were considered time-dependent variables, which were updated every 3 months. Serum calcium and phosphate levels were categorized into 3 groups: low (< 8.5 mg/dL [< 2.12 mmol/L], < 4.0 mg/dL [< 1.29 mmol/L]), medium (≥ 8.5–< 9.5 mg/dL [≥ 2.12–< 2.37 mmol/L], ≥ 4.0–< 7.0 mg/dL [≥ 1.29–< 2.26 mmol/L]), and high (≥ 9.5 mg/dL [≥ 2.37 mmol/L], ≥ 7.0 mg/dL [≥ 2.26 mmol/L]), respectively. The medium range for serum calcium and serum phosphate was defined based on the positive stratification for mortality in the previous report.^6^ Serum iPTH levels were grouped into < 300 or ≥ 300 pg/mL [9]. In this study, we examined the effect of serum calcium or serum phosphate on clinical outcomes, depending on the level of iPTH. Therefore, serum calcium and phosphate were categorized into 6 classes (3 levels of serum calcium or phosphate by 2 levels of iPTH).

When albumin levels were < 4.0 g/dL, serum calcium levels were corrected for albumin concentration by the modified Payne method [10] (which is commonly used in Japan): corrected calcium = calcium + (4.0 − albumin). Serum whole PTH levels measured with a third-generation PTH assay were converted to iPTH levels: iPTH = whole PTH × 1.7 [3].

Covariates included fixed patients’ characteristics (age, sex, primary kidney disease, diabetes, dialysis duration, cardiovascular disease, pulmonary disease, liver disease, malignancy, and history of parathyroidectomy) and time-dependent variables that were updated at each visit (MBD-related drugs coded as follows: VDRAs, oral/intravenous/none; phosphate binders, calcium carbonate/non–calcium-containing drugs/both/none; and calcimimetics, yes/no], serum albumin level, hemoglobin level, body mass index, Kt/V, and dialysate calcium concentration). In Japan, calcimimetics (cinacalcet hydrochloride) became available in January 2008.

### Statistical analysis

To estimate the average causal effect of MBD markers on mortality, we used marginal structural models [11, 12] to account for time-dependent confounders such as MBD treatments, by weighting with the inverse of the probability of having a history of a pattern of identifiable MBD markers.

For each 3-month period ending at visit *t*, the incidence of a clinical outcome was modeled based on the patterns of MBD markers in the previous 3 months (visit *t* − 1). The probability of having a pattern of identifiable MBD markers was calculated using pooled multinomial logistic regression models, in which the dependent variable was the pattern of MBD marker at visit *t* − 1 and the independent variables were time-dependent covariates at visit *t* − 2 and the baseline covariates mentioned above. Stabilized weights, in which the patterns of MBD markers at visit *t* − 2 were used as the numerator, were computed.

We used weighted Poisson regression to estimate adjusted incidence rates (aIRs) and adjusted incidence rate ratios (aIRRs). The weights were calculated as a cumulative product of the stabilized weights. Data were truncated if the cumulative stabilized weight was greater than 100 or less than 0.01. Periods of follow-up of cases in the subcohort before death and subcohort controls were further weighted by the inverse of the sampling fraction (1/0.4 = 2.5), while periods of follow-up in which the outcome events occurred were not (because the sampling fraction of cases was 1.0) [13, 14]. Within-patient correlation was assessed by using robust variances with an independent working correlation matrix. As sensitivity analysis, serum iPTH levels were grouped into < 240 or ≥ 240 pg/mL (sensitivity analysis 1 for serum calcium, and sensitivity analysis 3 for serum phosphate), serum calcium levels were categorized into 3 groups: low (< 8.4 mg/dL [< 2.10 mmol/L]), medium (≥ 8.4–< 10.0 mg/dL [≥ 2.10–< 2.49 mmol/L]), and high (≥ 10.0 mg/dL [≥ 2.49 mmol/L]) (sensitivity analysis 2), and serum phosphate levels were categorized into 3 groups: low (< 3.5 mg/dL [< 1.13 mmol/L]), medium (≥ 3.5–< 6.0 mg/dL [≥ 1.13–< 1.94 mmol/L]), and high (≥ 6.0 mg/dL [≥ 1.94 mmol/L]) (sensitivity analysis 4).

The results were summarized as point estimates and 95% confidence intervals (95% CIs) with *P* values. *P* values for interaction were also computed. SAS version 9.4 (SAS Institute Inc., Cary, NC) was used for all analyses.

## Results

### Baseline characteristics and outcomes

Table 1 shows the characteristics of the study patients summarized by visits per 3 months. Median age was 62 years and 62% of the patients were male. Median duration of dialysis was 10.2 years (interquartile range 3.8–14.4). The underlying disease was chronic glomerulonephritis in 45% of patients. Most patients had been prescribed medications to treat MBD: intravenous VDRAs in 53% of the total visits and phosphate binders in 86%. Calcimimetics, which were not available in Japan at the beginning of this study, were prescribed in 20% of visits. The all-cause and cardiovascular disease-related mortality rate was 4.9 and 1.8 per 100 person-years, respectively.

**Table 1:** Baseline patient characteristics of the study population

Characteristic	Value
Sex, male	62%
Age (years)	62 (55–71)
Dialysis duration (years)	10.2 (3.8–14.4)
Body mass index (kg/m^2^)	21.2 (18.8–23.1)
Serum albumin (g/dL)	3.7 (3.5–3.9)
Hemoglobin (g/dL)	10.5 (9.8–11.2)
Intact parathyroid hormone (pg/mL)	206 (124–320)
Calcium (mg/dL)	9.5 (9.0–10.0)
Phosphate (mg/dL)	5.4 (4.5–6.2)
Cause of end-stage renal disease	
Glomerulonephritis	45%
Diabetic nephropathy	25%
Comorbidities	
Cardiovascular disease	61%
Malignancy	5%
History of parathyroidectomy	6%
Vitamin D receptor activators	
Intravenous	53%
Oral	30%
None	17%
Phosphate binders	
Calcium-based	40%
Non-calcium-based	20%
Both	26%
None	14%
Cinacalcet	20%

Data are expressed as median (interquartile range) or proportion

According to the characteristics of subgroups by iPTH and calcium (Table S1), patients with higher iPTH levels tended to have been on dialysis for longer and prescribed intravenous VDRA more frequently. These trends became more apparent as serum calcium level increased. When the subgroups were analyzed by iPTH and phosphate, those patients with higher serum phosphate levels were more likely to be younger and receiving a VDRA (Table S2).

### Mortality and serum calcium levels stratified by serum iPTH levels

**Fig. 1:**
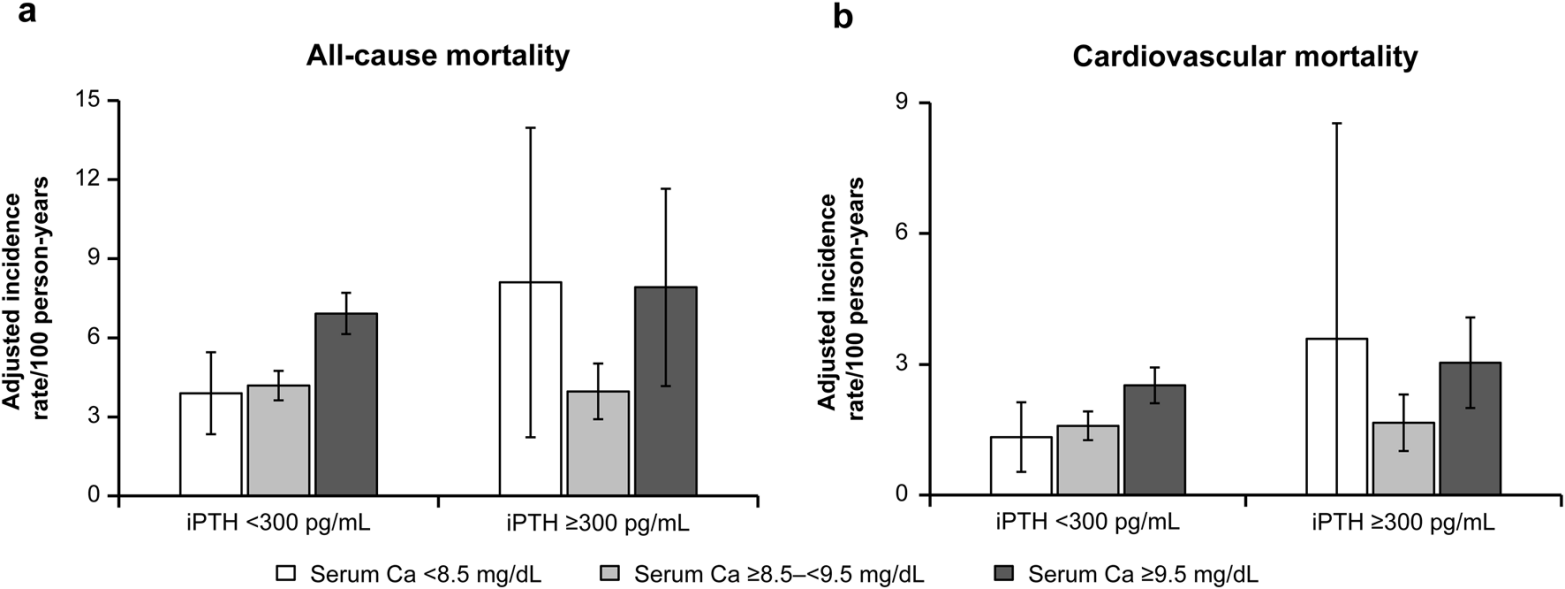
Adjusted incidence rate for all-cause mortality (**a**) and cardiovascular mortality (**b**) stratified by levels of serum intact parathyroid hormone and serum calcium. *Ca* calcium, *iPTH* intact parathyroid hormone. Incidence rate ratios were adjusted for patients’ characteristics (age, sex, primary kidney disease, diabetes, dialysis duration, cardiovascular disease, pulmonary disease, liver disease, malignancy, and history of parathyroidectomy) and time-dependent variables (vitamin D receptor activators, phosphate binders, calcimimetics, serum albumin level, hemoglobin level, body mass index, Kt/V, and dialysate calcium concentration)

Among the group with iPTH < 300 pg/mL, all-cause mortality rates in patients with high calcium levels (≥ 9.5 mg/dL) were higher than those in patients with medium levels (aIRR 1.65, 95% CI 1.39–1.96, *P* < 0.001) (Fig. 1, Tables 2, S3). However, among those with iPTH ≥ 300 pg/mL, we observed a U-shaped pattern in the aIRs. The interaction effect was marginally insignificant (*P* = 0.119). The aIRs increased not only among patients with high serum calcium levels but also among those with lower levels (Fig. 1). Similar trends were also observed regarding cardiovascular disease-related mortality, although the CIs were wider due to the small number of events (Fig. 1, Tables 2, S3).

**Table 2:** Adjusted incidence rate ratios for all-cause and cardiovascular mortality for patients with serum calcium levels stratified by serum intact parathyroid hormone levels

Outcomes	Serum calcium (mg/dL)	aIRR (95% CI)		Interaction
		iPTH < 300 pg/mL	iPTH ≥ 300 pg/mL	
All-cause mortality	< 8.5	0.93 (0.61–1.41)	2.04 (0.94–4.42)	*P* = 0.119
		*P* = 0.740	*P* = 0.070	
	≥ 8.5–< 9.5	Ref	Ref	
	≥ 9.5	1.65 (1.39–1.96)	1.99 (1.16–3.42)	
		*P* < 0.001	*P* = 0.012	
Cardiovascular mortality	< 8.5	0.84 (0.44–1.57)	2.16 (0.52–9.03)	*P* = 0.366
		*P* = 0.580	*P* = 0.293	
	≥ 8.5–< 9.5	Ref	Ref	
	≥ 9.5	1.58 (1.22–2.06)	1.83 (1.09–3.07)	
		*P* < 0.006	*P* = 0.0230	

Incidence rate ratios were adjusted for patients’ characteristics (age, sex, primary kidney disease, diabetes, dialysis duration, cardiovascular disease, pulmonary disease, liver disease, malignancy, and history of parathyroidectomy) and time-dependent variables (vitamin D receptor activators, phosphate binders, calcimimetics, serum albumin level, hemoglobin level, body mass index, Kt/V, and dialysate calcium concentration)

*aIRR* adjusted incidence rate ratio, *CI* confidence interval, *iPTH* intact parathyroid hormone, *ref* reference

In the sensitivity analysis, all-cause mortality rate in patients with high calcium levels (≥ 9.5 mg/dL) were higher than those in patients with medium levels in each iPTH level (cutoff value of iPTH 240 pg/mL), and the interaction effect was significant (*P* = 0.085) (Fig. S1, Table S4). Among the group with iPTH < 300 pg/mL, all-cause mortality rate in patients with low serum calcium level (< 8.4 mg/dL) and high serum calcium level (≥ 10.0 mg/dL) were higher than those in patients with medium levels, and the interaction effect was significant (*P* = 0.041) (Fig. S2, Table S5).

### Mortality and serum phosphate levels stratified by serum iPTH levels

**Fig. 2:**
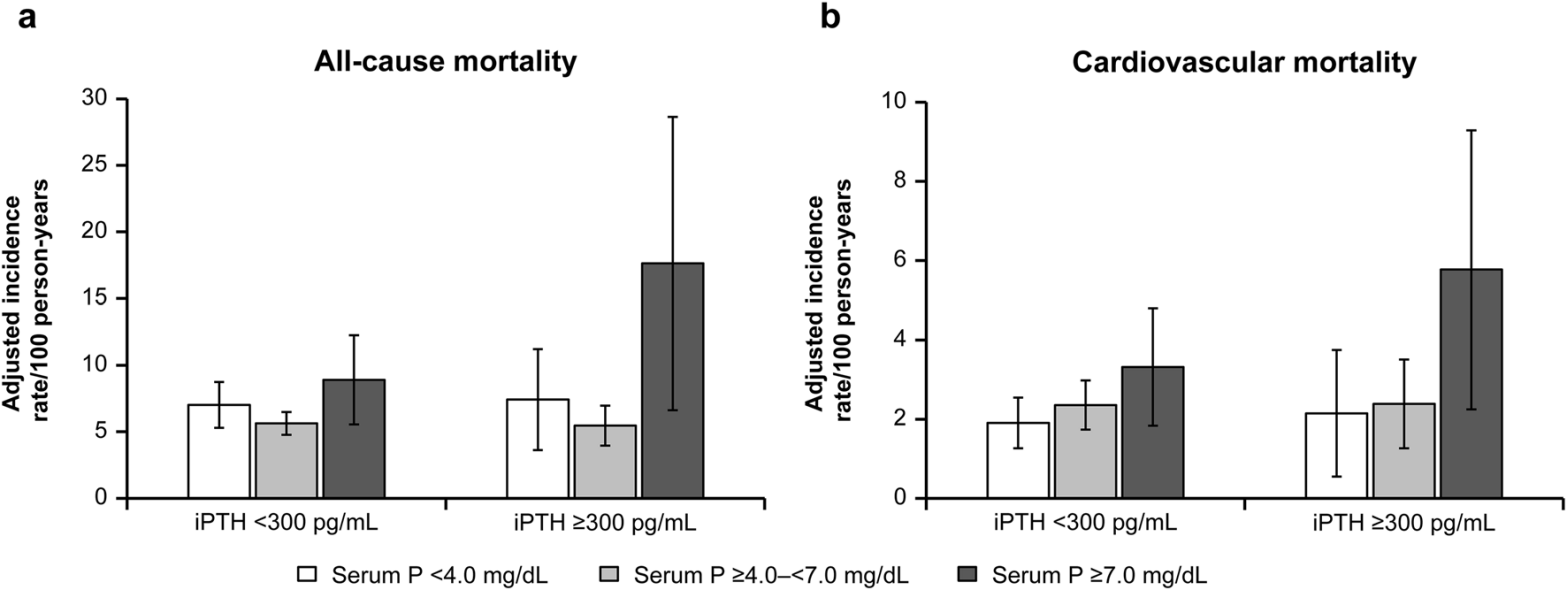
Adjusted incidence rate for all-cause mortality (**a**) and cardiovascular mortality (**b**) stratified by levels of serum intact parathyroid hormone and serum phosphate. *iPTH* intact parathyroid hormone, *P* phosphate. Incidence rate ratios were adjusted for patients’ characteristics (age, sex, primary kidney disease, diabetes, dialysis duration, cardiovascular disease, pulmonary disease, liver disease, malignancy, and history of parathyroidectomy) and time-dependent variables (vitamin D receptor activators, phosphate binders, calcimimetics, serum albumin level, hemoglobin level, body mass index, Kt/V, and dialysate calcium concentration)

The aIRs were higher among patients with high serum phosphate levels (Fig. 2). This pattern was more apparent when serum iPTH level was higher: the aIRR (95% CI) among patients with serum phosphate ≥ 7.0 mg/dL was 1.58 (1.06–2.36) when serum iPTH was < 300 pg/mL, and 3.23 (1.63–6.39) when serum iPTH was ≥ 300 pg/mL (Fig. 2, Tables 3, S6). The interaction effect was not statistically significant. The aIRs of cardiovascular disease-related mortality showed a similar trend (Fig. 2, Table S6). There was no significant interaction effect on clinical outcomes between serum iPTH and serum phosphate levels (Table 3).

**Table 3:** Adjusted incidence rate ratios for all-cause and cardiovascular mortality for patients with serum phosphate levels stratified by serum intact parathyroid hormone levels

Outcomes	Serum phosphate (mg/dL)	aIRR (95% CI)		Interaction
		iPTH < 300 pg/mL	iPTH ≥ 300 pg/mL	
All-cause mortality	< 4.0	1.25 (0.94–1.66)	1.36 (0.76–2.42)	*P* = 0.207
		*P* = 0.129	*P* = 0.298	
	≥ 4.0–< 7.0	Ref	Ref	
	≥ 7.0	1.58 (1.06–2.36)	3.23 (1.63–6.39)	
		*P* = 0.026	*P* < 0.001	
Cardiovascular mortality	< 4.0	0.81 (0.53–1.24)	0.90 (0.37–2.17)	*P* = 0.366
		*P* = 0.329	*P* = 0.815	
	≥ 4.0–< 7.0	Ref	Ref	
	≥ 7.0	1.41 (0.84–2.36)	2.41 (1.11–5.21)	
		*P* = 0.192	*P* = 0.025	

*aIRR* adjusted incidence rate ratio, *CI* confidence interval, *iPTH* intact parathyroid hormone, *ref* reference

Incidence rate ratios were adjusted for patients’ characteristics (age, sex, primary kidney disease, diabetes, dialysis duration, cardiovascular disease, pulmonary disease, liver disease, malignancy, and history of parathyroidectomy) and time-dependent variables (vitamin D receptor activators, phosphate binders, calcimimetics, serum albumin level, hemoglobin level, body mass index, Kt/V, and dialysate calcium concentration)

In the sensitivity analysis, all-cause mortality rate and cardiovascular morality rate in patients with high phosphate levels (≥ 6.0 mg/dL) were consistently higher than those in patients with medium levels in each iPTH level (cutoff value of iPTH 240 pg/mL) (Fig. S3, Table S7). Among the group with iPTH < 300 pg/mL, all-cause mortality rate in patients with high serum phosphate level (≥ 6.0 mg/dL) were attenuated (Fig. S4, Table S8).

## Discussion

This analysis showed that in maintenance hemodialysis patients with SHPT, severely affected patients, with iPTH ≥ 300 pg/mL and with high or low calcium levels (U shape), tended to have higher mortality rates, whereas in mildly affected patients with iPTH < 300 pg/mL, the mortality rates were higher only in patients with high calcium levels. Analysis by phosphate level showed that both severely and mildly affected patients with high phosphate levels had higher mortality rates; however, no significant difference was found with low phosphate levels.

With regard to the relationship between calcium levels and prognosis in hemodialysis patients, high calcium levels have consistently been shown to be associated with increased mortality rates [4–7]. Reports focusing on the relationship between calcium levels and prognosis depending on PTH level include those from the WW-DOPPS and United States Renal Data System (USRDS) studies. The WW-DOPPS study reported that patients with either high or low calcium levels had high mortality rates, both in the subpopulation with iPTH ≥ 300 pg/mL and that with iPTH < 300 pg/mL [4]. A study by Block et al. using the USRDS assessed the relationship between calcium levels and mortality rates or combined events of death and cardiovascular hospitalization by iPTH level, showing that the risk increased with high calcium levels (≥ 10 mg/dL) compared with corrected calcium levels in the control target range (8.4–10.0 mg/dL) at all iPTH levels, but not showing any change in the risk with low calcium levels (< 8.4 mg/dL) [15].

In our study, the risk of death increased significantly with high calcium levels (≥ 9.5 mg/dL) at all iPTH levels, and tended to increase with low calcium levels (< 8.5 mg/dL) in the iPTH ≥ 300 pg/mL stratum, but not in the < 300 pg/mL stratum. In the subpopulation with calcium levels < 8.5 mg/dL, patients with iPTH ≥ 300 pg/mL compared with those with iPTH < 300 pg/mL were characterized by shorter duration of hemodialysis, a higher proportion of untreated cases of VDRA, a higher male:female ratio, and a higher incidence of diabetic nephropathy. The aIRs for all-cause mortality and cardiovascular mortality in the subpopulation with iPTH ≥ 300 pg/mL and serum calcium < 8.5 mg/dL were more than double those in the subpopulation with iPTH < 300 pg/mL and serum calcium level < 8.5 mg/dL (Table S1). The poor prognosis for patients with iPTH ≥ 300 pg/mL and serum calcium < 8.5 mg/dL is attributable to any possible unmeasured confounding factors associated with the poor prognosis, as well as to the limitations on cinacalcet prescriptions and the inability to use VDRA optimally. Hypocalcemia may have strongly affected the worse prognosis in SHPT patients with iPTH ≥ 300 pg/mL and serum calcium < 8.5 mg/dL. In these patients, it seems necessary to use a calcimimetic and VDRA in combination in order to control mineral parameters appropriately.

With regard to the relationship between phosphate levels and prognosis in hemodialysis patients, this analysis, in line with previous studies [4–7], demonstrated increased mortality and cardiovascular deaths due to high phosphate levels. With regard to the relationship between low phosphate levels and mortality, an exhaustive survey in Japan^7^ showed increased risk of death with low phosphate levels (< 3.5 mg/dL), whereas a study by Block et al. [5] and a report by Fukagawa et al. from the MBD-5D study [6] found no increased mortality rates due to low phosphate levels. In our study, the risk of death did not change with low phosphate levels (< 4.0 mg/dL) in either of the iPTH strata (≥ 300 pg/mL and < 300 pg/mL). The risks with low phosphate levels might be attenuated by multi-adjusted factors such as undernutrition and frailty, which patients with low phosphate levels have.

This study has several strengths. First, the MBD-5D study was performed as a prospective case-cohort study, which enabled detailed and repeated data collection with few missing data and powerful outcome evaluations avoiding systematic biases. Second, participants of this study were restricted to hemodialysis patients with SHPT, who were generally at risk of abnormal MBD parameters. Therefore, the results of this study can be applied to patients requiring treatment for CKD-MBD.

The study has several limitations. First, it is not possible to measure unknown confounding factors. Second, it is unknown whether the causes of deviation of calcium and phosphate levels from the medium level categories are due to the natural disease course, medication, or other reasons; therefore, it is unclear whether the outcome varies depending on the reason for the parameter deviation. Third, in the study, we collected data on MBD parameters and MBD treatment every 3 months, and the changes that might occur during the 3-month periods were unknown; the influence of any such changes on outcomes therefore remains unknown. Fourth, the duration of observational periods and number of outcomes of each subgroup were not sufficient to analyze the effect modifications. Therefore, further study is required to confirm our results. Fifth, we performed the time-dependent analyses for 3-month intervals to evaluate the relationship between MBD parameters and mortality, these analyses might not predict the longer-term outcomes adequately.

## Conclusion

In patients with SHPT on hemodialysis, high calcium and phosphate levels were found to be associated with increased risk of death irrespective of the iPTH level.

## Electronic supplementary material

Below is the link to the electronic supplementary material.

Supplementary file1 (DOCX 59 kb)

Supplementary file2 (TIFF 184 kb)

Supplementary file3 (TIF 188 kb)

Supplementary file4 (TIF 166 kb)

Supplementary file5 (TIF 165 kb)
