# Supplementary material for: Relationship between serum calcium or phosphate levels and mortality stratified by parathyroid hormone level: an analysis from the MBD-5D study
Source: Clin Exp Nephrol. 2020 Mar 31;24(7):630–7. doi: 10.1007/s10157-020-01879-8 (PMC7271007; doi:10.1007/s10157-020-01879-8)
Supplement: Supplementary file 1 — Supplementary file1 (DOCX 59 kb) [file 10157_2020_1879_MOESM1_ESM.docx]

**Table S1.** Baseline patient characteristics and mortality of the total population and subgroups according to the time-dependent levels of intact parathyroid hormone and calcium

|  | iPTH <300 pg/mL | | | iPTH ≥300 pg/mL | | | | |
| --- | --- | --- | --- | --- | --- | --- | --- | --- |
|  | Ca <8.5 mg/dL | Ca ≥8.5–<9.5 mg/dL | Ca ≥9.5 mg/dL | |  | Ca <8.5 mg/dL | Ca ≥8.5–<9.5 mg/dL | Ca ≥9.5 mg/dL |
| Observational period, person-years | 1389 | 7592 | 9679 | |  | 639 | 2474 | 3456 |
| Sex, male | 73% | 63% | 60% | |  | 70% | 61% | 56% |
| Age, years | 65 (57–73) | 64 (56–73) | 63 (54–71) | |  | 62 (54–71) | 61 (53–70) | 62 (55–70) |
| Dialysis duration, years | 4.3 (1.9–9.3) | 5.8 (2.5–11.4) | 9.2 (4.7–15.9) | |  | 5.1 (2.0–10.6) | 8.3 (4.0–14.1) | 11.6 (7.7–17.4) |
| Body mass index, kg/m^2^ | 21.0 (19.0–23.3) | 20.9 (18.8–23.1) | 20.7 (18.8–23.0) | |  | 20.8 (19.0–23.7) | 21.1 (18.9–23.7) | 20.6 (18.7–22.7) |
| Serum albumin, g/dL | 3.8 (3.6–4.0) | 3.7 (3.5–3.9) | 3.7 (3.4–3.9) | |  | 3.8 (3.6–4.0) | 3.8 (3.6–4.0) | 3.7 (3.5–3.9) |
| Hemoglobin, g/dL | 10.5 (9.8–11.2) | 10.5 (9.9–11.2) | 10.6 (9.9–11.3) | |  | 10.6 (9.8–11.4) | 10.5 (9.8–11.2) | 10.5 (9.7–11.2) |
| Phosphate, mg/dL | 5.3 (4.4–6.1) | 5.2 (4.4–6.0) | 5.3 (4.5–6.2) | |  | 5.7 (4.7–6.9) | 5.7 (4.8–6.6) | 5.7 (4.9–6.5) |
| Cause of end-stage renal disease |  |  |  | |  |  |  |  |
| Glomerulonephritis | 31% | 39% | 48% | |  | 33% | 45% | 56% |
| Diabetic nephropathy | 41% | 31% | 22% | |  | 35% | 22% | 16% |
| Comorbidities |  |  |  | |  |  |  |  |
| Cardiovascular disease | 65% | 61% | 61% | |  | 61% | 59% | 60% |
| Malignancy | 5% | 5% | 4% | |  | 6% | 5% | 5% |
| History of parathyroidectomy | 6% | 4% | 6% | |  | 7% | 6% | 6% |
| Vitamin D receptor activators |  |  |  | |  |  |  |  |
| Intravenous | 30% | 46% | 59% | |  | 33% | 49% | 66% |
| Oral | 38% | 36% | 30% | |  | 27% | 24% | 16% |
| None | 32% | 18% | 11% | |  | 39% | 27% | 18% |
| Phosphate binders |  |  |  | |  |  |  |  |
| Calcium-based | 46% | 45% | 39% | |  | 40% | 37% | 32% |
| Non–calcium-based | 11% | 15% | 22% | |  | 13% | 19% | 30% |
| Both | 21% | 25% | 28% | |  | 26% | 28% | 25% |
| None | 22% | 15% | 11% | |  | 21% | 16% | 13% |
| Cinacalcet | 14% | 19% | 23% | |  | 17% | 18% | 17% |
| Mortality, per 100 person-years |  |  |  | |  |  |  |  |
| Crude incident rate |  |  |  | |  |  |  |  |
| All-cause mortality | 3.5 | 4.2 | 5.9 | |  | 3.9 | 3.4 | 5.3 |
| Cardiovascular mortality | 1.2 | 1.6 | 2.1 | |  | 1.2 | 1.4 | 2.2 |
| Adjusted incidence rate |  |  |  | |  |  |  |  |
| All-cause mortality | 3.90 (3.35, 5.46) | 4.19 (3.63, 4.75) | 6.92 (6.14, 7.69) | |  | 8.10 (2.23, 13.97) | 3.97 (2.91, 5.03) | 7.91 (4.17, 11.65) |
| Cardiovascular mortality | 1.33 (0.53, 2.13) | 1.59 (1.26, 1.92) | 2.52 (2.11, 2.94) | |  | 3.59 (-, 8.53) | 1.66 (1.01, 2.32) | 3.04 (2.00, 4.07) |

Total observation period was 25 229 person-years.

Data for patient characteristics are expressed as median (interquartile range) or proportion. Adjusted rates for mortality are expressed as point estimates (95% confidence intervals). Incidence rates were adjusted for patients’ characteristics (age, sex, primary kidney disease, diabetes, dialysis duration, cardiovascular disease, pulmonary disease, liver disease, malignancy, and history of parathyroidectomy) and time-dependent variables (vitamin D receptor activators, phosphate binders, calcimimetics, serum albumin level, hemoglobin level, body mass index, Kt/V, and dialysate calcium concentration).

Ca, calcium; iPTH, intact parathyroid hormone.

**Table S2.** Baseline patient characteristics and mortality of the total population and subgroups according to the time-dependent levels of intact parathyroid hormone and phosphate

|  | iPTH <300 pg/mL | | | iPTH ≥ 300 pg/mL | | | |
| --- | --- | --- | --- | --- | --- | --- | --- |
|  | P <4.0 mg/dL | P ≥4.0–<7.0 mg/dL | P ≥7.0 mg/dL |  | P <4.0 mg/dL | P ≥4.0–<7.0 mg/dL | P ≥7.0 mg/dL |
| Observational period, person-years | 2705 | 14 225 | 1729 |  | 493 | 4762 | 1314 |
| Sex, male | 60% | 62% | 68% |  | 55% | 59% | 64% |
| Age, years | 68 (59–76) | 63 (55–71) | 58 (49–66) |  | 66 (57–75) | 62 (55–71) | 58 (49–65) |
| Dialysis duration, years | 7.3 (3.0–13.7) | 7.8 (3.4–13.9) | 6.7 (2.9–11.5) |  | 9.9 (4.9–17.3) | 10.1 (5.7–16.1) | 8.9 (4.8–14.1) |
| Body mass index, kg/m^2^ | 20.4 (18.5–22.5) | 20.8 (18.9–23.1) | 21.4 (19.3–24.1) |  | 20.3 (18.3–22.5) | 20.8 (18.8–23.1) | 21.3 (19.2–24.0) |
| Serum albumin, g/dL | 3.6 (3.3–3.9) | 3.7 (3.5–3.9) | 3.8 (3.6–4.0) |  | 3.7 (3.4–3.9) | 3.7 (3.5–4.0) | 3.8 (3.5–4.0) |
| Hemoglobin, g/dL | 10.5 (9.7–11.2) | 10.6(9.9–11.3) | 10.6 (9.9–11.3) |  | 10.4 (9.7–11.1) | 10.5 (9.8–11.2) | 10.5 (9.7–11.3) |
| Calcium, mg/dL | 9.4 (8.9–9.9) | 9.4 (9.0–10.0) | 9.5 (8.9–10.0) |  | 9.5 (8.9–10.1) | 9.6 (9.0–10.2) | 9.5 (9.0–10.1) |
| Cause of end-stage renal disease |  |  |  |  |  |  |  |
| Glomerulonephritis | 40% | 44% | 39% |  | 51% | 51% | 46% |
| Diabetic nephropathy | 30% | 26% | 29% |  | 19% | 19% | 22% |
| Comorbidities |  |  |  |  |  |  |  |
| Cardiovascular disease | 66% | 60% | 59% |  | 70% | 59% | 58% |
| Malignancy | 7% | 4% | 3% |  | 9% | 5% | 4% |
| History of parathyroidectomy | 6% | 5% | 4% |  | 6% | 6% | 6% |
| Vitamin D receptor activators |  |  |  |  |  |  |  |
| Intravenous | 42% | 53% | 58% |  | 42% | 57% | 60% |
| Oral | 37% | 33% | 27% |  | 25% | 21% | 16% |
| None | 20% | 14% | 15% |  | 33% | 22% | 24% |
| Phosphate binders |  |  |  |  |  |  |  |
| Calcium-based | 49% | 42% | 35% |  | 40% | 36% | 30% |
| Non–calcium-based | 13% | 19% | 18% |  | 19% | 25% | 22% |
| Both | 18% | 26% | 37% |  | 15% | 24% | 37% |
| None | 20% | 13% | 10% |  | 26% | 14% | 11% |
| Cinacalcet | 17% | 21% | 21% |  | 17% | 17% | 17% |
| Mortality, per 100 person-years |  |  |  |  |  |  |  |
| Crude incident rate |  |  |  |  |  |  |  |
| All-cause mortality | 9.6 | 4.2 | 4.5 |  | 7.3 | 3.8 | 5.4 |
| Cardiovascular mortality | 2.8 | 1.6 | 2.0 |  | 2.2 | 1.6 | 2.2 |
| Adjusted incidence rate |  |  |  |  |  |  |  |
| All-cause mortality | 7.02 (5.30, 8.74) | 5.63 (4.77, 6.49) | 8.90 (5.55, 12.25) |  | 7.42 (3.62, 11.22) | 5.46 (3.96, 6.96) | 17.64 (6.63, 28.66) |
| Cardiovascular mortality | 1.91 (1.27, 2.55) | 2.36 (1.74, 2.97) | 3.32 (1.84, 4.80) |  | 2.15 (0.55, 3.76) | 2.39 (1.27, 3.52) | 5.77 (2.25, 9.29) |

Total observation period was 25 229 person-years.

Data for patient characteristics are expressed as median (interquartile range) or proportion. Adjusted rates for mortality are expressed as point estimates (95% confidence intervals). Incidence rates were adjusted for patients’ characteristics (age, sex, primary kidney disease, diabetes, dialysis duration, cardiovascular disease, pulmonary disease, liver disease, malignancy, and history of parathyroidectomy) and time-dependent variables (vitamin D receptor activators, phosphate binders, calcimimetics, serum albumin level, hemoglobin level, body mass index, Kt/V, and dialysate calcium concentration).

iPTH, intact parathyroid hormone; P, phosphate.

**Table S3.** Patients at risk and crude all-cause and cardiovascular mortality rate for patients with serum calcium levels stratified by serum intact parathyroid hormone levels

| Outcomes | Serum calcium (mg/dL) | Patients at risk and crude rate  [person-years (/100 person-years)] | |
| --- | --- | --- | --- |
|  |  | iPTH <300 pg/mL | iPTH ≥300 pg/mL |
| All-cause mortality | <8.5 | 1,389 (3.5) | 639 (3.9) |
|  | ≥8.5–<9.5 | 7,592 (4.2) | 2,474 (3.4) |
|  | ≥9.5 | 9,679 (5.9) | 3,456 (5.3) |
| Cardiovascular mortality | <8.5 | 1,401 (1.2) | 645 (1.2) |
|  | ≥8.5–<9.5 | 7,668 (1.6) | 2,492 (1.4) |
|  | ≥9.5 | 9,814 (2.1) | 3,496 (2.2) |

iPTH, intact parathyroid hormone

**Table S4.** Adjusted incidence rate ratios for all-cause and cardiovascular mortality for patients with serum calcium levels stratified by serum intact parathyroid hormone levels (Sensitivity Analysis 1)

| Outcomes | Serum calcium (mg/dL) | aIRR (95% CI) | | Interaction |
| --- | --- | --- | --- | --- |
|  |  | iPTH <240 pg/mL | iPTH ≥240 pg/mL |  |
| All-cause mortality | <8.5 | 0.69 (0.45–1.05) | 1.42 (0.79–2.55) | *P* = 0.085 |
|  |  | *P* = 0.085 | *P* = 0.244 |  |
|  | ≥8.5–<9.5 | ref | ref |  |
|  | ≥9.5 | 1.61 (1.34–1.95) | 2.33 (1.49–3.65) |  |
|  |  | *P* < 0.001 | *P* < 0.001 |  |
| Cardiovascular mortality | <8.5 | 0.74 (0.38–1.45) | 1.54 (0.50–4.78) | *P* = 0.142 |
|  |  | *P* = 0.384 | *P* = 0.455 |  |
|  | ≥8.5–<9.5 | ref | ref |  |
|  | ≥9.5 | 1.49 (1.11–2.00) | 2.57 (1.34–4.94) |  |
|  |  | *P* = 0.008 | *P* = 0.005 |  |

aIRR, adjusted incidence rate ratio; CI, confidence interval; iPTH, intact parathyroid hormone; ref,

reference.

Incidence rate ratios were adjusted for patients’ characteristics (age, sex, primary kidney disease, diabetes, dialysis duration, cardiovascular disease, pulmonary disease, liver disease, malignancy, and history of parathyroidectomy) and time-dependent variables (vitamin D receptor activators, phosphate binders, calcimimetics, serum albumin level, hemoglobin level, body mass index, Kt/V, and dialysate calcium concentration).

**Table S5.** Adjusted incidence rate ratios for all-cause and cardiovascular mortality for patients with serum calcium levels stratified by serum intact parathyroid hormone levels (Sensitivity Analysis 2)

| Outcomes | Serum calcium (mg/dL) | aIRR (95% CI) | | Interaction |
| --- | --- | --- | --- | --- |
|  |  | iPTH <300 pg/mL | iPTH ≥300 pg/mL |  |
| All-cause mortality | <8.4 | 0.77 (0.48–1.22) | 2.19 (1.11–4.35) | *P* = 0.041 |
|  |  | *P* = 0.266 | *P* = 0.025 |  |
|  | ≥8.4–<10.0 | ref | ref |  |
|  | ≥10.0 | 1.61 (1.32–1.97) | 2.02 (1.35–3.02) |  |
|  |  | *P* < 0.001 | *P* < 0.001 |  |
| Cardiovascular mortality | <8.4 | 0.64 (0.32–1.30) | 1.92 (0.54–6.84) | *P* = 0.085 |
|  |  | *P* = 0.220 | *P* = 0.314 |  |
|  | ≥8.4–<10.0 | ref | ref |  |
|  | ≥10.0 | 1.34 (0.98–1.84) | 2.50 (1.40–4.47) |  |
|  |  | *P* = 0.064 | *P* = 0.002 |  |

aIRR, adjusted incidence rate ratio; CI, confidence interval; iPTH, intact parathyroid hormone; ref, reference.

Incidence rate ratios were adjusted for patients’ characteristics (age, sex, primary kidney disease, diabetes, dialysis duration, cardiovascular disease, pulmonary disease, liver disease, malignancy, and history of parathyroidectomy) and time-dependent variables (vitamin D receptor activators, phosphate binders, calcimimetics, serum albumin level, hemoglobin level, body mass index, Kt/V, and dialysate calcium concentration).

**Table S6.** Patients at risk and crude all-cause and cardiovascular mortality rate for patients with serum phosphate levels stratified by serum intact parathyroid hormone levels

| Outcomes | Serum phosphate (mg/dL) | Patients at risk and crude rate  [person-years (/100 person-years)] | |
| --- | --- | --- | --- |
|  |  | iPTH <300 pg/mL | iPTH ≥300 pg/mL |
| All-cause mortality | <4.0 | 2,705 (9.6) | 493 (7.3) |
|  | ≥4.0–<7.0 | 14,225 (4.2) | 4,762 (3.8) |
|  | ≥7.0 | 1,729 (4.5) | 1,314 (5.4) |
| Cardiovascular mortality | <4.0 | 2,774 (2.8) | 502 (2.2) |
|  | ≥4.0–<7.0 | 14,363 (1.6) | 4,801 (1.6) |
|  | ≥7.0 | 1,745 (2.0) | 1,330 (2.2) |

iPTH, intact parathyroid hormone

**Table S7**. Adjusted incidence rate ratios for all-cause and cardiovascular mortality for patients with serum phosphate levels stratified by serum intact parathyroid hormone levels (Sensitivity Analysis 3)

| Outcomes | Serum phosphate  (mg/dL) | aIRR (95% CI) | | Interaction |
| --- | --- | --- | --- | --- |
|  |  | iPTH <240 pg/mL | iPTH ≥240 pg/mL |  |
| All-cause mortality | <4.0 | 1.51 (1.16–1.96) | 1.34 (0.92–1.94) | *P* = 0.797 |
|  |  | *P* = 0.002 | *P* = 0.123 |  |
|  | ≥4.0–<7.0 | ref | ref |  |
|  | ≥7.0 | 2.22 (1.24–4.00) | 1.86 (1.20–2.88) |  |
|  |  | *P* = 0.008 | *P* = 0.005 |  |
| Cardiovascular mortality | <4.0 | 1.37 (0.89–2.11) | 0.93 (0.46–1.89) | *P* = 0.475 |
|  |  | *P* = 0.152 | *P* = 0.847 |  |
|  | ≥4.0–<7.0 | ref | ref |  |
|  | ≥7.0 | 1.82 (1.05–3.14) | 2.45 (1.26–4.77) |  |
|  |  | *P* = 0.032 | *P* = 0.008 |  |

aIRR, adjusted incidence rate ratio; CI, confidence interval; iPTH, intact parathyroid hormone; ref, reference.

Incidence rate ratios were adjusted for patients’ characteristics (age, sex, primary kidney disease, diabetes, dialysis duration, cardiovascular disease, pulmonary disease, liver disease, malignancy, and history of parathyroidectomy) and time-dependent variables (vitamin D receptor activators, phosphate binders, calcimimetics, serum albumin level, hemoglobin level, body mass index, Kt/V, and dialysate calcium concentration).

**Table S8**. Adjusted incidence rate ratios for all-cause and cardiovascular mortality for patients with serum phosphate levels stratified by serum intact parathyroid hormone levels (Sensitivity Analysis 4)

| Outcomes | Serum phosphate  (mg/dL) | aIRR (95% CI) | | Interaction |
| --- | --- | --- | --- | --- |
|  |  | iPTH <300 pg/mL | iPTH ≥300 pg/mL |  |
| All-cause mortality | <3.5 | 1.45 (1.00–2.11) | 1.42 (0.64–3.18) | *P* = 0.996 |
|  |  | *P* = 0.051 | *P* = 0.389 |  |
|  | ≥3.5–<6.0 | ref | ref |  |
|  | ≥6.0 | 1.37 (0.99–1.90) | 1.34 (0.95–1.89) |  |
|  |  | *P* = 0.060 | *P* = 0.091 |  |
| Cardiovascular mortality | <3.5 | 1.36 (0.81–2.27) | 1.19 (0.24–5.98) | *P* = 0.383 |
|  |  | *P* = 0.240 | *P* = 0.832 |  |
|  | ≥3.5–<6.0 | ref | ref |  |
|  | ≥6.0 | 1.69 (1.17–2.44) | 1.11 (0.69–1.78) |  |
|  |  | *P* = 0.005 | *P* = 0.668 |  |

aIRR, adjusted incidence rate ratio; CI, confidence interval; iPTH, intact parathyroid hormone; ref, reference.

Incidence rate ratios were adjusted for patients’ characteristics (age, sex, primary kidney disease, diabetes, dialysis duration, cardiovascular disease, pulmonary disease, liver disease, malignancy, and history of parathyroidectomy) and time-dependent variables (vitamin D receptor activators, phosphate binders, calcimimetics, serum albumin level, hemoglobin level, body mass index, Kt/V, and dialysate calcium concentration).
