# Supplementary figures and images for: Relationship between serum calcium or phosphate levels and mortality stratified by parathyroid hormone level: an analysis from the MBD-5D study
Source: Clin Exp Nephrol. 2020 Mar 31;24(7):630–7. doi: 10.1007/s10157-020-01879-8 (PMC7271007; doi:10.1007/s10157-020-01879-8)

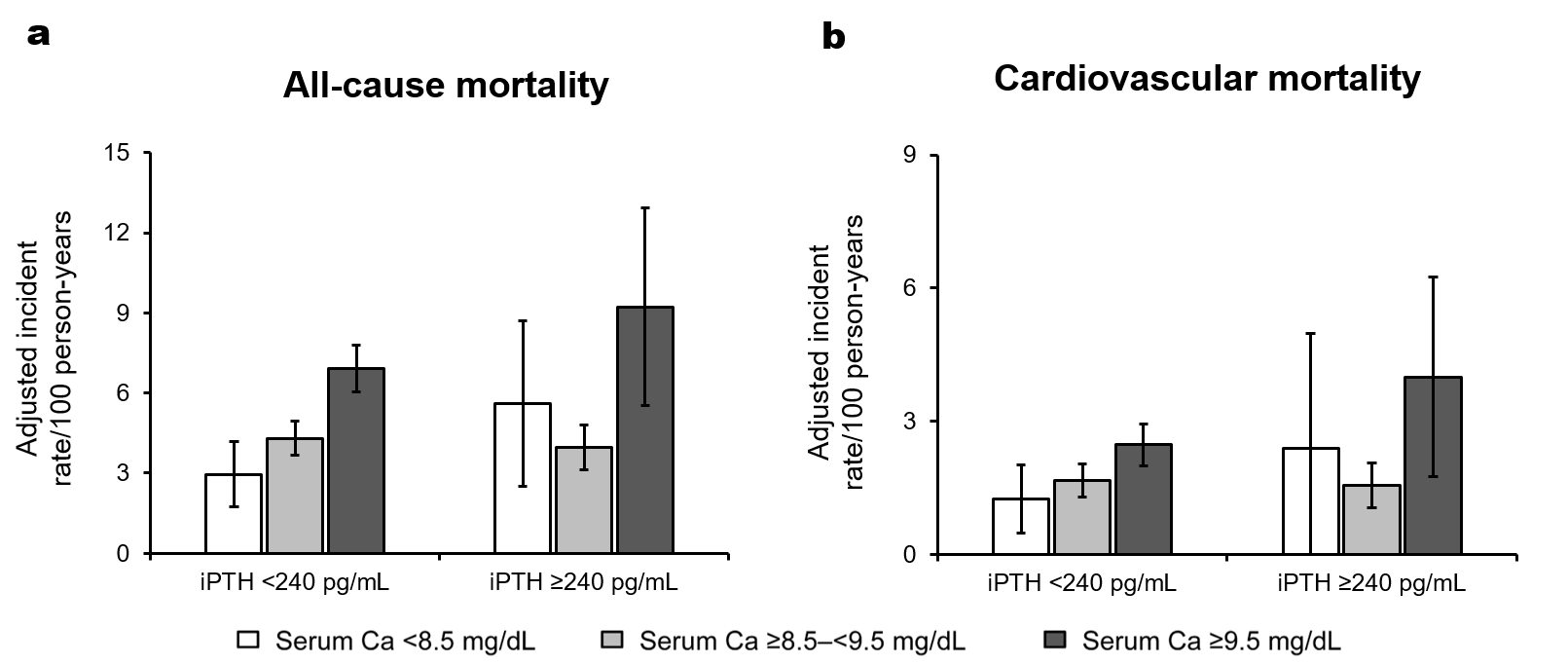

Supplement: Supplementary file 2 — Supplementary file2 (TIFF 184 kb) [file 10157_2020_1879_MOESM2_ESM.tif]

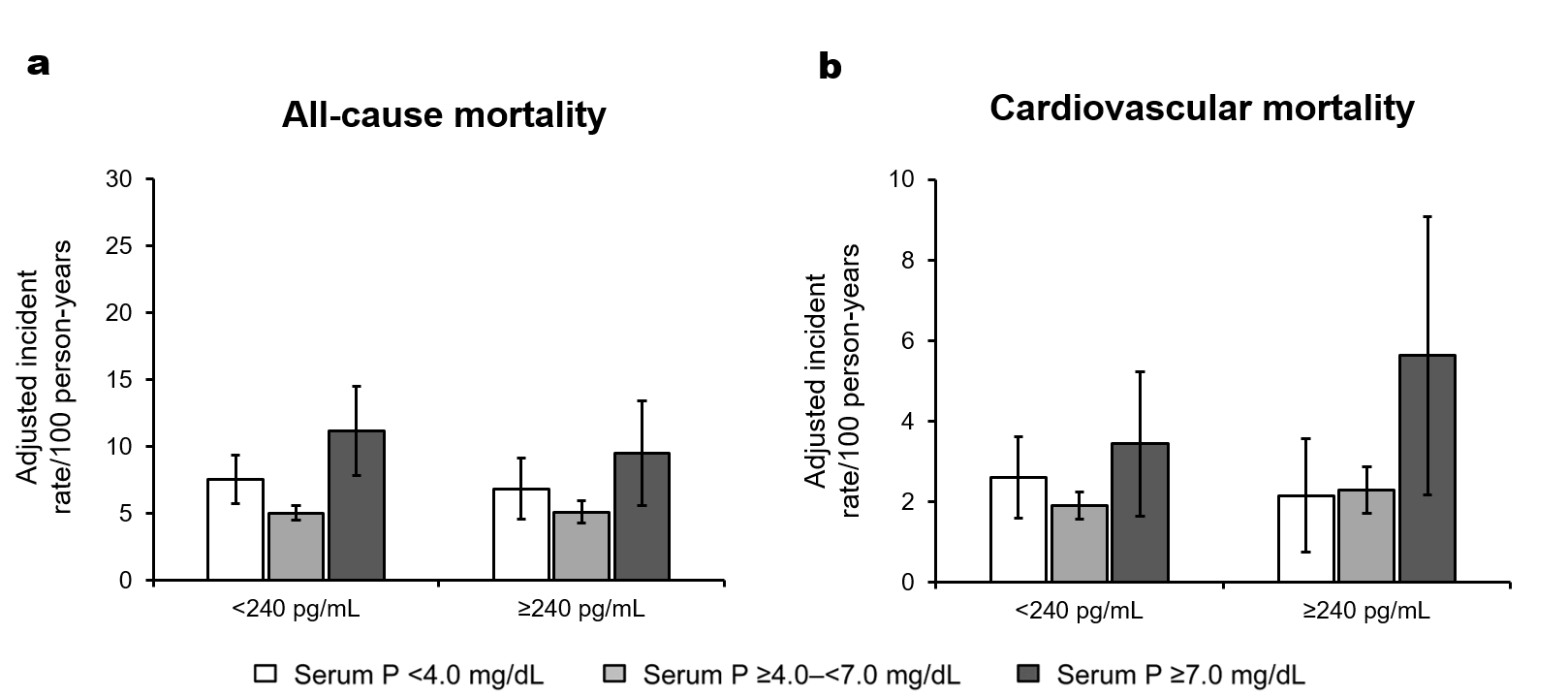

Supplement: Supplementary file 3 — Supplementary file3 (TIF 188 kb) [file 10157_2020_1879_MOESM3_ESM.tif]

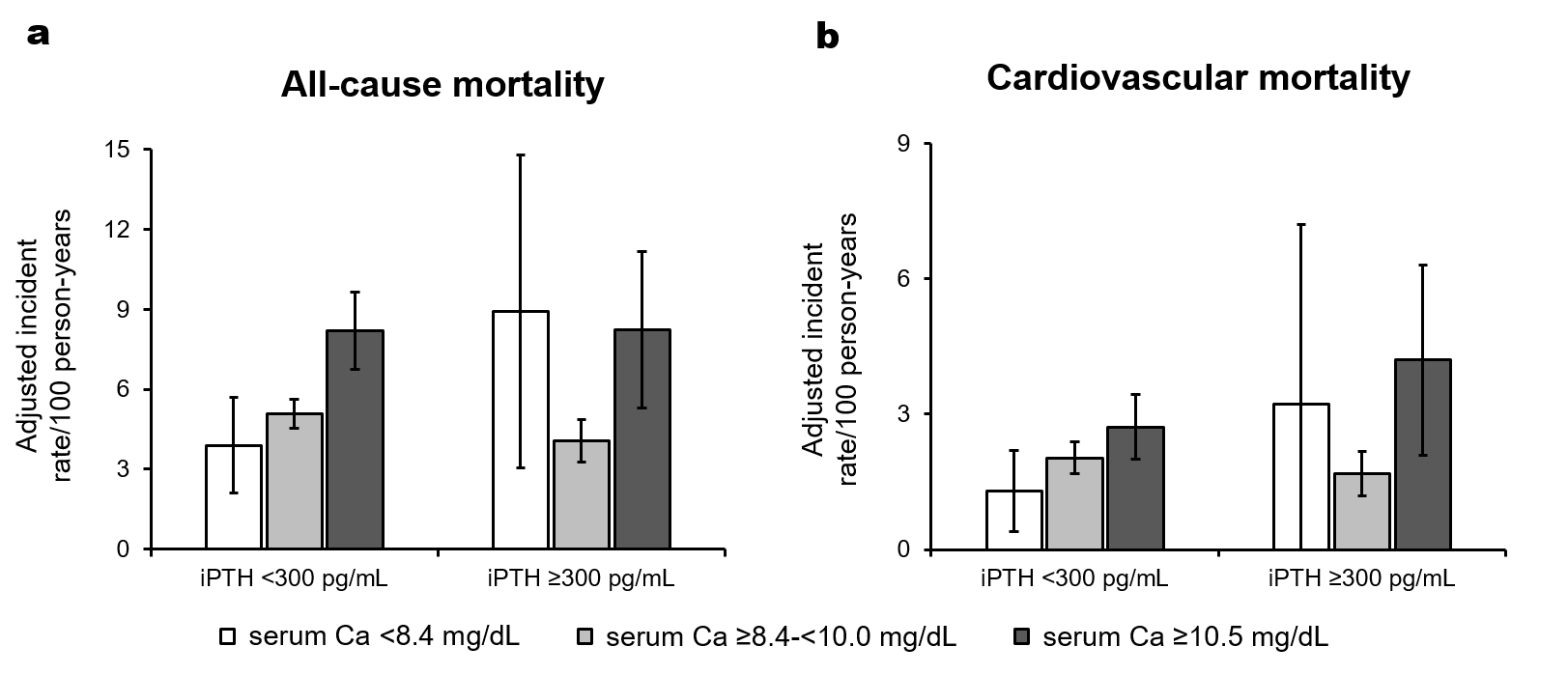

Supplement: Supplementary file 4 — Supplementary file4 (TIF 166 kb) [file 10157_2020_1879_MOESM4_ESM.tif]

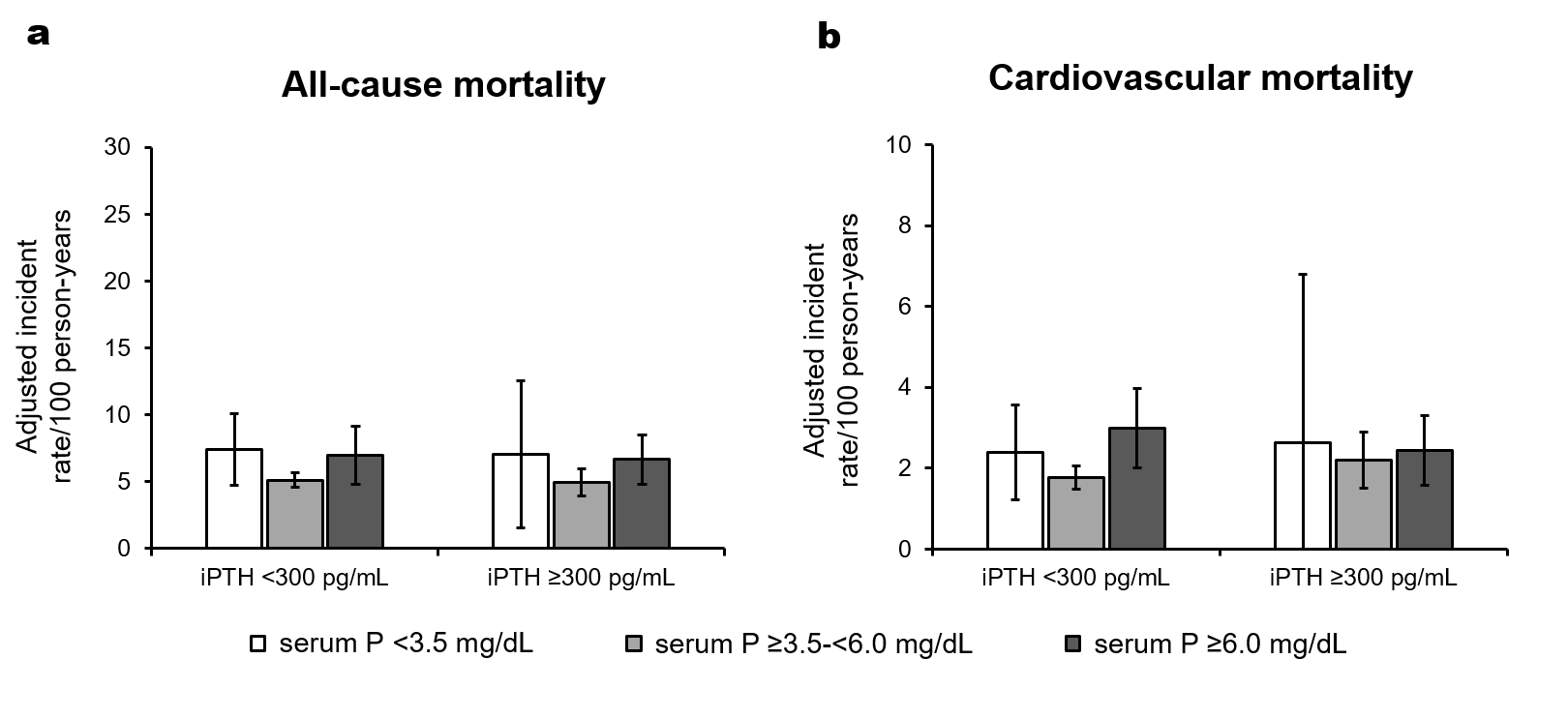

Supplement: Supplementary file 5 — Supplementary file5 (TIF 165 kb) [file 10157_2020_1879_MOESM5_ESM.tif]
